# Cultural Incentive Learning: How Culture Shapes Acquisition of Values

**DOI:** 10.1002/evan.70005

**Published:** 2025-07-01

**Authors:** Francesco Rigoli, Jack Lennon

**Affiliations:** ^1^ Department of Psychology City St Georges, University of London London UK; ^2^ School of Archaeology and Ancient History Leicester UK

**Keywords:** conditioned reinforcement, culture, decision‐making, goal‐directed, imitation, incentive, learning, value

## Abstract

Although research on human values is abundant, it has so far neglected a crucial question: what are the psychological mechanisms whereby culture shapes people's values? To address this, the manuscript introduces a framework examining how culture shapes the acquisition of values, a process referred to as *cultural incentive learning*. The proposal is that cultural incentive learning mediates the influence exerted by the structure of society upon people's values. According to the framework, when the social structure changes, certain forms of learning (i.e., conditioned reinforcement) are elicited which promote value change. Simultaneously, other forms of learning, which are based on imitating other people's behavior, pull toward the preservation of previous values, ensuring that value change is not too precipitous and that group cooperation is maintained. Applying these principles to cultural evolution, the paper develops a theory of how values evolve over history, a process we label *Value Evolution*.

## 1 Introduction

The notion of *value* is central to research in the social sciences. Indeed, various theories in this field have assumed that human behavior is rooted in decision‐making processes that aim at fulfilling values. Broadly speaking, research on how human values develop can be categorized into two threads. Focusing on the psychological level, the first line of enquiry has examined how learning mechanisms such as conditioned reinforcement [1, 2, 3, 4, 5] and social learning [6, 7] drive the acquisition of values. The second thread, instead, has analyzed the role of culture. Within the latter line of research, scholars have explored how values vary from culture to culture and how certain characteristics of society favor the emergence of some values at the expense of others [8, 9, 10, 11, 12, 13]. Theoretically, the role of culture in this domain has been explained by cultural evolution theory [14, 15, 16, 17, 18, 19], which examines how cultural traits, including those concerning values, evolve over history.

While the two research threads, the psychological and cultural ones, are thriving in their own right, attempts to integrate the two have been relatively few. We argue that this state of affairs is undesirable, and that substantial insight can be gained by integrating the psychological and cultural outlook on the question of how human values develop. Based on this consideration, the paper proposes a theoretical framework that embeds the psychological mechanisms underlying acquisition of values within cultural processes, and explores the implications for the study of cultural evolution.

The paper is structured over three parts. The first part overviews the psychological processes driving the acquisition of values. The second part introduces a theoretical framework that embeds the psychological processes within cultural dynamics. The last part examines the implication of this framework for cultural evolution theory.

## 2 Psychology

### 2.1 The Concept of Value

**Figure 1:**
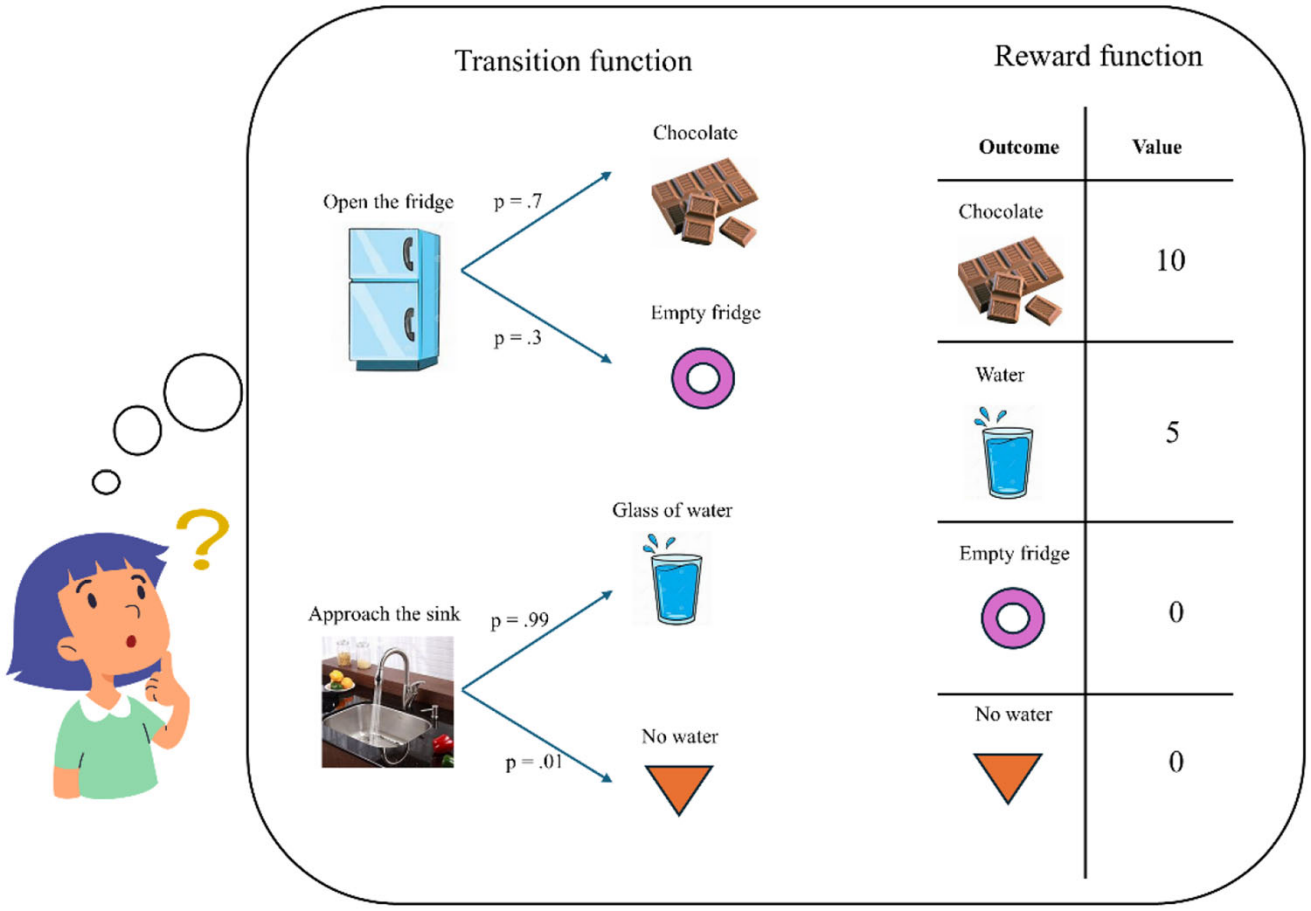
Schematic illustration of decision‐making according to dynamic programming, describing a scenario where a child has to make a choice between two available options—open the fridge and approach the sink. Here decision‐making is based on integrating two kinds of beliefs: the transition function, representing beliefs about action outcomes contingencies, and the reward function, representing beliefs about the value of outcomes.

Terms such as goal, value, reward, punishment, utility, and incentive are central to various social science disciplines. However, these terms are used inconsistently by different disciplines and even by competing theories within the same discipline. To clarify how we use these terms, here we rely on dynamic programming, which is the standard approach used in computer science and engineering to model decision‐making [20, 21]. Dynamic programming assumes that, at any point in time, our brain is capable of representing a *decision tree* by simulating different courses of action (Figure 1). By representing the consequences of a chain of actions, the decision tree can extend deep into the future. Moreover, by postulating some uncertainty about which outcome is produced by any action, it can be probabilistic. Any decision tree is constructed based on two sources of knowledge: the *transition function* and the *reward function*. The former corresponds to knowledge about the action‐outcome contingencies, and it can be expressed verbally by the proposition “if state *x* occurs at time *t*, then action *a* leads to state *y* at time *t* + 1 with probability *p.*” For example, if an actor is in the kitchen now, then opening the fridge leads to a chocolate bar with probability 0.9. Intuitively, the transition function describes a cognitive map of the environment and of the action rules at play to navigate it. The other element of the decision tree is the reward function. To each outcome represented within the decision tree, the reward function attaches a number which describes how desirable (when the number is positive) or undesirable (when it is negative) the outcome is. For example, the kitchen may be linked with indifference, thus being attached a value of 0, while the chocolate, being highly desirable, may be linked with a positive value of 10. Combining the transition function with the reward function is, within this framework, what determines choice behavior.

From this picture, we can define the key terms used in the paper. The term *value* indicates whether, and to what extent, an agent views an expected outcome as desirable (when its value is positive), undesirable (when its value is negative), or neutral (when its value is equal to 0), with values being predicated by the reward function. The term *reward* refers to expected outcomes which, according to the reward function, are linked with positive value. The term *punishment* refers to expected outcomes associated with a negative value. Collectively, rewards and punishments are referred to as *incentives*. Armed with these definitions, we can reformulate the question investigated in the paper as follows: what are the learning processes whereby culture shapes the formation of human incentives? Or, alternatively, what are the learning processes whereby culture imbues outcomes with value? We refer to this form of learning as *cultural incentive learning*. The paper investigates how cultural incentive learning works.

Note that the framework proposed above implies that an incentive is something desirable (for rewards) or undesirable (for punishments) as such; in other words, it is an *end* in and of itself, and not a *means* for something else. The distinction between means and ends is crucial here, since our focus is on studying how culture shapes the formation of human ends (i.e., something valuable as such), not of means (i.e., something valuable to achieve another outcome). In dynamic programming, the distinction between ends and means maps to the distinction between the reward function (predicating which outcomes are viewed as ends) and the transition function (predicating the means necessary to achieve such ends). According to this framework, therefore, asking how the reward function develops is equivalent to asking how human ends are formed, while asking how the transition function develops is equivalent to asking how knowledge about means is acquired. Based on this distinction, the paper focuses on the psychological processes driving the development of the reward function (not of the transition function) and investigates how these processes are molded by culture.

Now that the general psychological framework has been illustrated, we turn to exploring the origin of the reward function, in other words, we explore where incentives come from. We shall see that some incentives, which we label *innate incentives*, are established by evolution, while other incentives, which we label *acquired incentives*, are developed through experience via a process referred to as *incentive learning*.

### 2.2 Innate Incentives

A common assumption in psychology and biology is that, for humans, some outcomes are inherently associated with positive or negative value by evolutionary design [22]. These can be labeled as *innate rewards* (when they are associated with positive value) or *innate punishments* (when they are associated with negative value), and, collectively, as *innate incentives*. Alongside outcomes relevant for basic physiological needs such as food, water, sexual partners, and painful stimuli, research has shown that innate incentives also encompass outcomes linked with two basic human motives, that is, social [23] and cognitive motives (also referred to as intrinsic motivations [24]).

### 2.3 Conditioned Reinforcement

Alongside innate incentives, psychological research has documented two fundamental processes whereby previously neutral outcomes (i.e., outcomes which are not innate incentives to begin with) can garner value, thus becoming *acquired incentives*. The first of such processes is *conditioned reinforcement* [1, 3, 4, 5]. In a study on this [2], rats initially underwent a classical conditioning procedure where a neutral stimulus (a visual cue) was presented before food (sugar dissolved in water). At the end of the session, by eliciting a conditioned response (CR) of salivation, the visual cue had become a conditioned stimulus (CS). Next, animals were administered a chemical (lithium chloride) in conjunction with the food employed during classical conditioning. This manipulation is known to link food with sickness, hence devaluing food thereafter. In a test phase, animals were presented with a lever and, if they pressed it, they received the CS in the absence of food. The researchers observed that rats pressed the lever repeatedly to obtain the CS. Importantly, by devaluing food after classical conditioning, the experiment shows that, during the test phase, the CS was not sought because it was a means for obtaining food, but, rather, because it had become an end in and of itself. This reveals that, despite being associated with no value at the outset, the CS had acquired value during classical conditioning, thus becoming an *acquired reward*. This is an instance of an incentive whose value is not innate but learnt.

Various studies have revealed that conditioned reinforcement does not only occur when, during the classical conditioning phase, an innate reward or punishment is experienced directly, but also when it is experienced vicariously [25, 26, 27]. In one of these studies [25], children were divided into three groups: one (*direct reward group*) which, after performance of an appropriate action, was presented with a visual cue followed by reward; one (*observed reward group*) which observed other children who, after performance of an appropriate action, were presented with a visual cue followed by reward; and a control group including children who were not exposed to any cue nor to reward. A test phase revealed that, in comparison with the control group, both the direct reward group and the observed reward group were later more likely to perform an action leading to the visual cue, even in the absence of reward—supporting the idea that the cue had become an acquired reward also for the observed reward group. This and similar evidence [26, 27] has revealed the existence of *vicarious conditioned reinforcement*.

### 2.4 Imitative Incentive Learning

Alongside conditioned reinforcement, research has documented a second learning mechanism whereby previously neutral outcomes can become acquired incentives. This mechanism is grounded on the human propensity to imitate other people [23, 28]. In one study on this [6], 3–5‐year‐old children first performed a task where a correct response was followed by the reception of a plastic disc. Not surprisingly, at this stage, receiving the disc did not improve the children's performance, indicating that the disc was initially appraised as neutral. Next, the children observed peers who, while performing a task, received the plastic disc (the children could not observe the peers' behavior, but they observed delivery of the disc). In a test phase, the children were asked to perform another task where, once again, they received the disc following a correct response. The analyses revealed that, now, reception of the disc improved performance. This finding is consistent with the following interpretation. During the observation phase, children inferred that the peers' incentive was to obtain the disc. In turn, this inference spurred imitation, thereby motivating children to acquire the same incentive for themselves, in other words, to view the disc as valuable and therefore to pursue it. This explains why the children's performance improved when, in the test phase, it was rewarded with the disc. These and similar results [6, 7, 29] have highlighted a human propensity to infer the incentives pursued by other people and to appropriate those incentives for themselves. This represents a psychological process whereby initially neutral outcomes acquire value, a process which is distinct from conditioned reinforcement. Given the role played by imitation, we refer to this process as *imitative incentive learning*.

In the study of imitative incentive learning, a critical question is whether people imitate all targets equally or whether they display a preference to imitate some particular targets. The literature on imitation has revealed that a target is more likely to be imitated when possessing the following characteristics [30]: similarity with the observer, proficiency in the action performed, high status, and (especially for children) intention to teach. It can be conjectured, therefore, that people preferentially appropriate the incentives pursued by these targets.

We have now overviewed the psychological mechanisms that drive the acquisition of values. To recapitulate, empirical research has revealed that, alongside innate incentives, acquired incentives can be learnt via conditioned reinforcement and imitative incentive learning. Conditioned reinforcement occurs when a neutral stimulus is paired with an innate incentive. The consequence of such pairing is that the neutral stimulus garners value and thus becomes an acquired incentive. Conditioned reinforcement not only occurs with direct experience, but it can also occur vicariously, that is, by observing another person exposed to the neutral stimulus anticipating the innate incentive. Imitative incentive learning occurs when, after an agent has inferred the incentive pursued by another person, the agent appropriates this incentive for oneself. In light of the psychological research just overviewed, the next part embeds this psyhological research within a broader framework where cultural factors are also at play.

## 3 Culture

### 3.1 Cultural Incentive Learning Framework

Abundant evidence has documented how values vary from culture to culture and how certain characteristics of society favor the emergence of some values at the expense of others [8, 9, 10, 11, 12, 13, 31]. Still, research in this domain has neglected the specific psychological mechanisms whereby culture shapes people's values. To address this, here we develop a theoretical framework to explain how these psychological mechanisms are shaped by culture. As we shall see, our theory addresses questions such as: What are the incentives embraced by a person living in a certain cultural context? Which cultural factors determine these incentives? And which psychological processes drive their acquisition? To illustrate our theory, we will consider a concrete example, that of French serfdom in the High Middle Ages as described by the historian Bloch [32].

**Figure 2:**
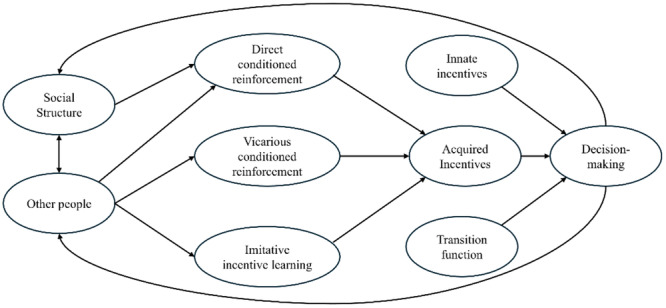
Graphical representation of the Cultural Incentive Learning Framework (CILF). Arrows represent causal influences.

**Figure 3:**
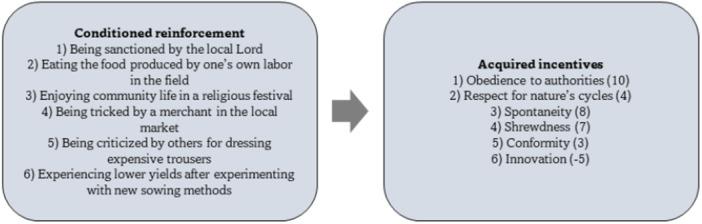
Hypothetical list of conditioned reinforcement experiences and ensuing acquired incentives for the French medieval serfdom example.

Our proposal, which we label Cultural Incentive Learning Framework (CILF), is illustrated in Figure 2. Each node of the framework represents a variable at play, with arrows describing causal influences among the variables (bidirectional arrows reflect bidirectional causal influences). The node at the core of the CILF describes the Acquired incentives characteristic of the agent (e.g., characteristic of a French medieval serf). Note that, while innate incentives are postulated to be invariant across cultures, in this framework acquired incentives are what distinguishes peoples and cultures, and therefore they are the key variable within the CILF. A way to represent these is via a list of outcomes which are treated as acquired incentives by the agent, with each outcome being linked with a number reflecting its value. Figure 3 provides a hypothetical list for the French medieval serf example (e.g., with one of the acquired incentives being obedience to authorities). The three nodes projecting to the Acquired incentives node represent learning experiences which, according to the CILF, shape acquired incentives. Depending on which mechanism is at play, three kinds of experiences can be envisaged: those linked with direct conditioned reinforcement, those linked with vicarious conditioned reinforcement, and those linked with imitative incentive learning, respectively. It is straightforward to describe experiences linked with imitative incentive learning: these simply occur when the agent observes another person pursuing an incentive (e.g., when the observed person complies with authorities' orders). Regarding experiences linked with conditioned reinforcement, these can be described by events leading to the collection of innate incentives. A hypothetical list of such experiences is provided in Figure 3 for the French medieval serf example. Here, for instance, the experience of being sanctioned by the local lord promotes obedience to authorities as an acquired incentive. Note that the list is applicable to direct and vicarious forms of conditioned reinforcement alike. The difference is that, in the former case, the experience is made personally, while in the latter case, the experience is vicarious (i.e., it is made by another person). Within a culture, important sources of vicarious conditioned reinforcement are tales, myths, and rumors circulating within the community. For instance, historians have documented the relevance of tales such as Little Red Riding Hood in terms of revealing the values (e.g., the importance of being shrewd) embraced by the masses in medieval and early modern France [33].

The nodes on the left of Figure 2 describe the factors eliciting the learning experiences responsible for the formation of acquired incentives. The first node, called Social Structure, refers to factors underlying the structure of society. What the concept of structure means in this context is left vague on purpose, as this is not the place for a systematic analysis of this aspect. Generally, all factors determining the agent's experience of the association between neutral stimuli and innate incentives pertain to the Social Structure node, thus encompassing, among others, geographical, technological, organizational, economic, institutional, and legal factors. The Social Structure node is proposed to influence the Direct conditioned reinforcement node. In the example of French medieval serfs, the Social Structure node captures the existence of a feudal system where serfs were expected to surrender part of the harvest to the lord and where the lord administrated local justice [32]. This, according to the CILF, causes the experience of being sanctioned by the local lord, which in turn promotes obedience to authorities as an acquired incentive.

The second node on the left, referred to as Other People, reflects the behavior performed by people from the different groups within a community (note that the model postulates a bidirectional causal influence between the Social Structure and Other People node, as reflected in the bidirectional arrow connecting the two). It is useful to isolate this node because this allows us to emphasize that vicarious conditioned reinforcement and imitative incentive learning are driven specifically by observing other people's actions. With this regard, it is important to stress that, according to empirical research, not all observed people are equally influential, but exert an influence which depends on aspects such as similarity, prestige, and status [30]. When this principle is applied to the cultural context, it is important to note that similarity, prestige, and status are not objective characteristics, but they are based on cultural assumptions [34]. For example, in the European Middle Ages, the different orders were believed to be established by birth, implying that people from other groups were perceived as very dissimilar [35]. On this basis, French serfs were probably more likely to imitate other serfs rather than imitating nobles or clergymen: despite the latter having higher status, they were nonetheless perceived as too distant. A very different pattern, arguably, characterizes Western societies today: although, for instance, enormous wealth differences persist among groups, the prevailing cultural discourse now views all citizens as equal (e.g., supported by evidence that the majority of US citizens categorize themselves as middle class [36]). This implies that, compared to the Middle Ages, now low‐status individuals may be more likely to imitate high‐status individuals.

The Acquired incentives node projects to the Decision‐making node. The framework also includes two other nodes projecting to the latter: the Innate Incentive and Transition function nodes. This simply means that, in combination with the transition function, acquired and innate incentives (collectively determining the reward function) underpin decision‐making behavior. Finally, Figure 2 depicts arrows projecting back from the Decision‐making node to the Social Structure and Other People nodes. This implies that, according to the CILF, acquired incentives guide people's decision‐making in such a way that eventually transforms society. Put another way, here cultural values are not an epiphenomenon without importance, but, following a rich tradition traced back at least to Max Weber's work [37], they are powerful forces driving social change.

### 3.2 Temporal Dynamics

The processes described so far ignore temporal dynamics. In other words, up to this point, the focus has been on the ongoing social structure, the ongoing learning experiences, and the ongoing acquired incentives. Yet all these variables change over time: the social structure changes because of factors internal to itself (e.g., an invasion of a foreign population or climatic changes) and because of the influence of the group's decision‐making behavior. In turn, by affecting learning experiences, changes in the social structure impact on the acquired incentives. Is it possible to extend the CILF to explain these temporal dynamics? One way to address this simply consists in postulating that the variables depicted in Figure 2 change over time; in other words, Figure 2 can be interpreted as describing a dynamical system. This allows one to probe how the incentives embraced by a certain culture or group have changed over generations. For example, it can address questions such as: Why has a certain community moved away from valuing martial virtues to praising commercial attitudes, or vice versa? Or why has religious piety spread so much within a certain culture?

In general, the CILF envisages two different pathways that can lead to value change within a group. The difference between the two pathways concerns whether the group's actions play a critical role in reshaping the group's values or not. The first pathway, which can be called the *passive pathway*, occurs when the social structure is modified because of its internal dynamics, for instance following a foreign invasion, a pandemic outbreak, a regime change, or natural disasters. Here the group's behavior plays little to no role in modifying the social structure. Following the structural modifications, the learning experiences made by the group are reshaped, and this in turn changes the group's acquired incentives. Several instances of such passive pathway can be identified throughout history. For example, some historians have argued that, after the Black Death reduced the European population by one‐third (a very substantial structural change of which the peasants were obviously not responsible), the bargaining power of peasants vis‐à‐vis their landlords grew greatly [38, 39, 40]. This, according to these historians, led peasants to covet freedom and autonomy (an instance of newly acquired incentives), as evidenced by their frequent revolts during the decades following the Black Death, ultimately leading to the abolition of serfdom in many jurisdictions [38, 39, 40].

The second pathway leading to value change, which can be called *active pathway*, occurs when the group's behavior plays an important role in modifying the social structure. Once the social structure is modified, the process is the same as for the passive pathway: new learning experiences occur, leading, over time, to value change. Once again, history offers many examples of active pathways. For instance, Weber [37] famously proposed that a key role in forging the institutions of early capitalism (a substantial social structural change) was played by the behavior of the European Protestant bourgeoisie, driven by religious values prescribing thrift and wealth accumulation. Following this line of reasoning, some have argued that, once the capitalist system was established, this in turn promoted radically new acquired incentives within the Protestant bourgeoisie itself, for instance, the desire for free trade and for luxury goods, and the praise of innovation [41, 42].

We have concluded our overview of the CILF. We now proceed by examining the implications of this framework for cultural evolution theory. As we shall see, applying the CILF in this domain offers various insights, including insight on how values evolve over history.

## 4 Cultural Evolution

The last part of the paper examines the CILF in the context of research on cultural evolution. The analysis unfolds over five subsections. The first summarizes the literature on cultural evolution. The second assesses the CILF in the context of previous theories in the field that have focused on payoff biases. The third assesses the CILF in the context of cultural evolution theories that have focused on context‐based social learning strategies. The fourth speculates on the possible evolutionary functions of cultural incentive learning. Finally, the fifth subsection adopts the CILF to analyze the processes whereby the values embraced by a population evolve over history.

### 4.1 Background Literature

Among scholars, there is growing interest in the role played by culture during the evolutionary history of the human species [14, 15, 16, 17, 18, 19]. In this debate, culture is defined as a set of psychological contents (e.g., beliefs, norms, values, practices, and skills) that vary between and within human populations and are transmitted from one generation to the next via social learning. An influential proposal is that the human species is unique insofar as it has evolved culture to adapt to rapidly changing environments [19]. According to this view, the advantage of culture is that, compared to natural selection, it enables a population to adapt much more rapidly to a new environment, thus explaining why the human species manifests such a striking ability to thrive in the most various ecological settings. This raises the question of which mechanisms are responsible for cultural adaptation. Drawing a parallel with natural selection, two processes have been envisaged [17]: one creating variation during cultural transmission, the other responsible for the selection of certain cultural contents over others. There is debate on how these processes work, especially concerning the nature of cultural selection [14, 15, 16, 17, 18, 19, 43]. An influential view [44, 45] is that cultural selection is rooted in innate biases encompassing *content‐based biases* (whereby some cultural traits are inherently more attractive than others) and *context‐based biases* (that depend on contextual aspects such as on whether the source of the cultural content is prestigious or familiar). Moreover, certain cultural contents diminish in frequency and eventually disappear simply because they impair the organism's fitness, that is, because they hamper the production of heirs [17]. By contrast, other cultural contents proliferate by promoting fitness. In the long run, culture can even drive natural selection: the classical example is the fact that certain human populations, but not others, today have genes enabling digestion of lactose in adulthood, presumably because their ancestors developed a culture where dairy food was regularly consumed ([15]; but see the study of Evershed et al. [46], who have recently questioned this interpretation).

### 4.2 Cultural Incentive Learning and Payoff Bias

The literature just outlined raises the question of whether the CILF can contribute to explain aspects of cultural evolution. Relevant here is the proposal that, among the innate cognitive biases responsible for the selection of cultural traits, an important one is based on selecting traits that maximize genetically determined payoffs (e.g., food) [47, 48, 49, 50, 51]. In turn, the underlying assumption is that genetically determined payoffs have been selected by evolution for their significance for the species' fitness. For example, a specific arrow design (an instance of a cultural trait) may be selected at the expense of an alternative design since it leads to catching more games and therefore to more food (an instance of a genetically determined payoff). A recent formulation of this idea has been made by Singh [51], who has applied it to explain disparate cultural phenomena including technology, magic, ritual, esthetic traditions, and institutions.

Along these lines, some scholars [47, 52] have distinguished between *genetically determined payoffs* and *intermediate payoffs*, with the assumption that intermediate payoffs (e.g., arrow aiming accuracy) are instrumental to achieve genetically determined payoffs (e.g., food). According to this proposal (which we shall call Intermediate Payoff Model), people can develop a causal theory of the relationship between intermediate payoffs and genetically determined payoffs and, once this causal theory is in place, they can select a cultural trait (e.g., arrow design) just based on maximizing the intermediate payoff (e.g., arrow aiming accuracy), disregarding the genetically determined payoff (e.g., food).

In some respects, the Intermediate Payoff Model has similarities with the CILF. However, the psychological assumptions made by the two frameworks are substantially different. Indeed, the Intermediate Payoff Model presupposes that, psychologically, intermediate payoffs are viewed as means to achieve ends (with the latter corresponding to genetically determined payoffs), and not as ends in and of themselves. Employing the language of dynamic programming, the Intermediate Payoff Model presupposes that, according to the reward function, intermediate payoffs have no value. They are sought only because, based on the transition function, they are instrumental to achieve genetically determined payoffs (the latter, instead, are assumed to be linked with value). Put another way, for the Intermediate Payoff Model, all incentives are ultimately innate, not acquired. By contrast, as discussed at length above, a central claim of the CILF is that previously neutral outcomes can become valuable in and of themselves, thus becoming acquired incentives. In short, while the Intermediate Payoff Model does not contemplate the existence of acquired incentives, the CILF stresses the role played by them. These considerations highlight how the CILF may contribute to research in this domain. Specifically, the CILF encourages research on how payoffs drive cultural evolution to acknowledge explicitly the role played by acquired incentives.

### 4.3 Cultural Incentive Learning and Context‐Based Social Learning Strategies

The CILF is also relevant for research investigating how social learning strategies drive cultural evolution [44, 45]. In this literature, an influential taxonomy differentiates between two sets of strategies [45]:

– *Content‐based strategies* (aka “what” strategies), in which social learning depends on the content of the behavior to imitate. For instance, if the observed behavior is appealing as such (e.g., it is pleasurable), or if it leads to an appealing outcome (e.g., a person may copy a certain hunting technique because the technique works better), then the behavior will be more likely to be copied.
– *Context‐based strategies*, where social learning is activated based on contextual features such as internal states (e.g., if a person is uncertain, then social learning is more likely to occur [53]) or certain characteristics of the model (e.g., prestige [54] and familiarity), as in model‐based, or “who,” strategies.

Based on this taxonomy, the CILF may contribute to research exploring the role played by incentive learning during context‐based strategies. At present, it remains unclear whether, and in which situations, context‐based strategies recruit incentive learning. Take the prestige bias (i.e., people's tendency to imitate prestigious models) [54], which is an instance of context‐based strategy. Imagine a case where a person has copied the haircut of a prestigious model. Here the question is: Why has the person imitated the prestigious model's haircut? Does this occur because the person has ended up liking the haircut as such? In other words, does it occur because the haircut has become an end in and of itself (it has become an acquired incentive)? Or, rather, because the person views the haircut as means to be liked by others (thus becoming prestigious herself)? The latter possibility implies that the haircut is not liked as such, but it is adopted as means to achieve prestige—the haircut, in other words, is not an acquired incentive. As this example illustrates, at present it remains unknown whether, and in which situations, context‐based strategies like the prestige bias recruit incentive learning. The CILF encourages scholars to explore this question empirically.

Another aspect of cultural evolution which is relevant for the CILF concerns the mechanisms underlying cultural transmission. For instance, Derex et al. [55] studied cultural transmission in the lab by examining how the techniques used to solve a problem are passed over multiple “generations” of participants (see Harris et al. [56] for a similar study conducted in the field). The study reports that, while the techniques employed to solve the problem become more effective over generations, participants' causal knowledge of why the techniques work remain limited. An interesting question raised by the CILF is whether incentive learning plays any role in cultural transmission processes like those described by Derex et al. [55]. The paradigm used by the authors leaves this question open since the processes that may underpin incentive learning cannot be isolated from other factors. The CILF encourages scholars to design paradigms that can isolate the role of incentive learning during cultural transmission.

Note that Derex et al. [55] show that participants have poor causal knowledge about why their techniques work (see also Harris et al. [56]). Does this demonstrate that participants have acquired new incentives? We argue that this is not the case. Indeed, a possibility is that the participants' end was simply to maximize task performance, and that this end remained unchanged during the task—implying that incentive learning was not involved. This possibility is fully compatible with the evidence documented by Derex et al. [55]. Indeed, it is possible to interpret the study's findings as showing that participants learnt that certain techniques were better for maximizing performance, even though they had no idea of why those techniques worked. Note that this implies that they learnt about contingencies in the environment (i.e., that certain techniques led to better performance), even without knowledge of the causes of these contingencies. This form of learning does not require incentive learning (it requires a form of learning which, in the context of dynamic programming, concerns the transition function). We believe that the scenario we have just described, which does not require any incentive learning, is plausible. This implies that the fact that participants did not acquire causal knowledge does not imply that incentive learning was engaged.

In conclusion, we have discussed the implications of the CILF for research in cultural evolution concerning the payoff bias, social learning strategies, and cultural transmission. Generally speaking, the potential contribution of the CILG to these topics is to introduce an explicit formulation of the concept of acquired incentive, which is new to the field of cultural evolution. Note that being a new concept does not mean that the phenomenon described is particularly surprising or unexpected. Intuitively, for example, it makes sense to imagine that, at least sometimes, social learning (and especially context‐based strategies) may lead to the formation of acquired incentives, and there is nothing particularly surprising in proposing such hypothesis. Still, an explicit formulation of the concept of acquired incentive is absent in cultural evolution theory, and introducing such concept can offer at least two benefits. First, as discussed here, it can inspire empirical research on cultural evolution that aims to isolate the impact of incentive learning. Second, it can offer insight into how human values evolve, a question we explore later.

### 4.4 The Evolutionary Function of Cultural Incentive Learning

To appraise the CILF in the context of research on cultural evolution, an important question concerns the evolutionary function of cultural incentive learning. Up to this point, the CILF has been developed inductively, starting from empirical evidence and attempting to identify the underlying principles. This raises the question of whether these principles can be interpreted as fulfilling any evolutionary function.

Regarding conditioned reinforcement, we argue that its evolutionary function can be ascribed to simplifying decision‐making in terms of the cognitive resources employed. Indeed, less resources are needed if, rather than learning the complex causal relationships between neutral stimuli and innate incentives (such as between a visual cue and food), the brain treats some neutral stimuli (those that typically anticipate innate incentives) as valuable as such, something which is realized by conditioned reinforcement. The evolutionary cost of this strategy is that it is suboptimal in terms of maximizing innate incentives (e.g., the environment may change in such a way that an acquired incentive does not anticipate an innate incentive anymore). Yet, the benefit is that cognitive resources can be saved. On balance, benefits may prevail over costs, explaining why conditioned reinforcement has emerged as an evolutionary strategy.

Regarding imitative incentive learning, its evolutionary function is arguably the same function played by imitation in general. Two aspects have been identified by the literature on this topic [15, 17]. One concerns the fact that imitation allows the integration of knowledge acquired by multiple individuals, enlarging the total cognitive resources dedicated to learning and enabling cumulative culture. The second evolutionary function of imitation is to ensure some level of conformity, which in turn fosters cooperation. These two functions, it can be argued, characterize imitative incentive learning as much as other forms of imitation. Indeed, if a person appropriates the incentives sought by others rather than learning incentives alone, then the person is more likely to develop acquired incentives that, on average, are adaptive (i.e., acquired incentives that lead to innate incentives). At the same time, when the people of a community share their incentives, they are more likely to cooperate to achieve those incentives.

One last question is worth considering here: to what extent are incentive learning processes unique to the human species, and to what extent are they shared also by other animals? There is incontrovertible evidence that conditioned reinforcement characterizes all mammals and, possibly, also other animal classes [5]. Yet, it is possible that, compared to other animals, the level of sophistication of conditioned reinforcement is higher in humans [43]. This enhanced sophistication may consist in a higher number of acquired incentives and in a more abstract definition of acquired incentives. While direct conditioned reinforcement is not a human exclusivity, there is no evidence of vicarious conditioned reinforcement nor of imitative incentive learning outside the human species, though systematic comparative research on this remains to be carried out. This supports the idea that certain complex forms of social learning are uniquely human [23].

### 4.5 Value Evolution

The last aspect of cultural evolution that we shall discuss concerns how values evolve within a population. Before conducting this analysis, though, an important clarification is in order. Most literature in the field has presupposed that the same learning processes underpin the evolution of different cultural features such as values, beliefs, norms, practices, and skills. Although this is a reasonable approximation to be made at an early stage of the enquiry, it becomes less compelling as research progresses, since the psychological literature has revealed that different learning processes underlie the formation of different mental representations. On this basis, we stress that our analysis is restricted to values and not to other cultural features such as beliefs, practices, or skills. Using once more dynamic programming as framework, we examine how the reward function characterizing a population evolves, not how the transition function does so. In other words, we do not deal with the question of how a population develops knowledge about new means to achieve its ends (e.g., how it develops more efficient hunting techniques to maximize food). Rather, we enquire about how the population's values (i.e., incentives) evolve—we refer to this form of cultural evolution as *Value Evolution*. To our knowledge, an analysis with a specific focus on Value Evolution remains to be carried out.

Our argument begins by noting that, according to the CILF, changes in the social structure are the forces spurring value change. Indeed, according to the CILF, changes in the social structure imply that some people within a population start making novel conditioned reinforcement experiences and thus develop novel acquired incentives. While novel conditioned reinforcement experiences push toward value change, imitative incentive learning may often pull toward the conservation of previous values. The reason is that, as illustrated above, imitative incentive learning leads to conformity with the majority. If, after a change in the social structure has occurred, most people have not yet updated their incentives thanks to conditioned reinforcement, then imitative incentive learning will typically resist value change. This occurs because most people still embrace the old values and because, as just said, imitative incentive learning typically leads to conformity with the majority. The implication of this clash between conditioned reinforcement and imitative incentive learning (the former pushing toward value change and the latter pulling for value conservation) can produce a period of value conflict within a population, with the members more amenable to conditioned reinforcement striving for change and those more prone to imitative incentive learning resisting change. Note that, typically, the scenario will be even more complex than the one just sketched, because the value conflict will often modify the social structure further, with a cascade of added consequences.

A historical example where these processes may have unfolded is the dawn of the Industrial Revolution in England. Despite offering unprecedented economic opportunities for employers, the introduction of innovative technologies in the textile industry was initially resisted by many who followed the more conservative values typical of the town guilds [57, 58]. Following the CILF, we can speculate that a value conflict arose between a smaller number of employers who, based on new conditioned reinforcement experiences, were now embracing the new values of innovation and efficiency, versus a larger number of employers who, based on imitative incentive learning (i.e., based on imitating the majority), stick with the old values of tradition and regulation.

Interestingly, the conservative role played by imitative incentive learning may explain why, within a culture, certain values sometimes persist even when they are not supported anymore by conditioned reinforcement. This may apply to some rituals and taboos which are vigorously enforced by a culture despite having, both from an external and internal point of view, no obvious function. Although these taboos and rituals may have initially developed because of conditioned reinforcement, at some point, the latter might have ceased to be a factor. Nevertheless, especially if pursuing them is not too demanding, these rituals and taboos may still be preserved by the culture thanks to the role played by imitative incentive learning (see [52] for a similar argument).

The picture just described raises the question of whether, according to the CILF, Value Evolution is ultimately adaptive. To address this question, remember that changes in the social structure elicit novel conditioned reinforcement experiences. These, in turn, allow a population to tune its acquired incentives to the new circumstances, in such a way that access to innate rewards is maintained (remember that, based on conditioned reinforcement, acquired incentives typically anticipate innate incentives). In turn, within the CILF, innate rewards are postulated to be established by natural selection because of their importance in supporting the organism's fitness. Thus, the CILF implies that, insofar as it ultimately promotes fitness, value change spurred by new conditioned reinforcement experiences is adaptive. Still, from an evolutionary perspective, value change is also risky since it requires abandoning acquired incentives that have worked relatively well so far. For the group, therefore, it is adaptive to employ a strategy which ensures that value change is not too precipitous but occurs at the right pace. Imitative incentive learning may be such a strategy. Indeed, by leveraging on a sort of wisdom‐of‐the‐crowd mechanism, imitative incentive learning may slow down value change, hence ensuring that this proceeds in a balanced way. Imitative incentive learning, moreover, may have the added benefit of preserving group cohesion during changes of the social structure, thereby ensuring that cooperation among the group's members is upheld. In short, the picture emerging from the CILF is one where conditioned reinforcement (by spurring value change) and imitative incentive learning (by resisting value change) work together to ensure that, as the environment is transformed, the population's values change in a balanced way and cooperation is maintained.

## 5 Conclusion

To conclude, the manuscript raises an important question which has rarely been considered by previous literature in the social sciences: How do psychological and cultural processes interact in the formation of human values? To address this question, the manuscript starts by examining psychological research that documents two fundamental types of incentive learning: (direct and vicarious) conditioned reinforcement and imitative incentive learning. On this basis, the manuscript introduces a framework where these psychological processes are embedded within cultural dynamics and explores the implications of this framework for cultural evolution theory. The proposal is that specific aspects of the social structure determine the conditioned reinforcement and imitative incentive learning experiences gathered by a social group, which, in turn, shape the groups' values. When, due to its internal dynamics or to the group's decision‐making, the social structure changes, then new conditioned reinforcement experiences ensue, which in turn drive value change. At the same time, imitative incentive learning may pull toward the preservation of previous values, ensuring that value change is not too precipitous and that group cooperation is maintained. The balance between conditioned reinforcement (which pulls toward value change) and imitative incentive learning (which resists change) is, according to our framework, what ultimately drives the evolution of values over history.

The paper offers a first attempt to develop a theory of cultural incentive learning that may inspire theoretical debate and empirical enquiry on such an important, yet often neglected, research topic.

## Ethics Statement

The authors have nothing to report.

## Consent

The authors have nothing to report.

## Conflicts of Interest

The authors declare no conflicts of interest.

## Data Availability

The authors have nothing to report.
